# Parametric Analysis of Electrical Conductivity of Polymer-Composites

**DOI:** 10.3390/polym11081250

**Published:** 2019-07-29

**Authors:** Oladipo Folorunso, Yskandar Hamam, Rotimi Sadiku, Suprakas Sinha Ray, Adekoya Gbolahan Joseph

**Affiliations:** 1 French South African Institute of Technology (F’SATI)/Department of Electrical Engineering, Tshwane University of Technology, Pretoria 0001, South Africa; 2 École Supérieure d’Ingénieurs en Électrotechnique et Électronique, Cité Descartes, 2 Boulevard Blaise Pascal, Noisy-le-Grand, 93160 Paris, France; 3 Institute of Nanoengineering Research (INER)/Department of Chemical, Metallurgy and Material Engineering, Tshwane University of Technology, Pretoria 0001, South Africa; 4 DST-CSIR National Centre for Nanostructured Materials, Council for Scientific and Industrial Research, Pretoria 0001, South Africa; 5 Department of Chemical Sciences, University of Johannesburg, Doornfontein, Johannesburg 2028, South Africa

**Keywords:** polymers, fillers, models, percolation, electrical, conductivity

## Abstract

The problem associated with mixtures of fillers and polymers is that they result in mechanical degradation of the material (polymer) as the filler content increases. This problem will increase the weight of the material and manufacturing cost. For this reason, experimentation on the electrical conductivities of the polymer-composites (PCs) is not enough to research their electrical properties; models have to be adopted to solve the encountered challenges. Hitherto, several models by previous researchers have been developed and proposed, with each utilizing different design parameters. It is imperative to carry out analysis on these models so that the suitable one is identified. This paper indeed carried out a comprehensive parametric analysis on the existing electrical conductivity models for polymer composites. The analysis involves identification of the parameters that best predict the electrical conductivity of polymer composites for energy storage, viz: (batteries and capacitor), sensors, electronic device components, fuel cell electrodes, automotive, medical instrumentation, cathode scanners, solar cell, and military surveillance gadgets applications. The analysis showed that the existing models lack sufficient parametric ability to determine accurately the electrical conductivity of polymer-composites.

## 1. Introduction

The use of formulated mathematical equations to design, investigate, and predict physical, or chemical state of a process, based on the properties of systems, is called modeling. Models are products of scientific investigation formulated into equations which can be solved analytically or numerically [1]. The promising state of polymer-composites (PCs) will obviously take the entire globe to the next level of technological growth, if properly researched. Fuel cell energy may quickly replace fossil fuel in powering turbines and machineries, if adequate and relevant researches are focussed on conductive polymer-composites (CPCs). This conductive material can be easily manufactured, and it is relatively inexpensive [2,3]. In order to utilize the versatile advantages of PCs, the essence of modeling cannot be overemphasized in order to predict its electrical conductivity and mechanical strength. Improving the electrical conductivity of PCs is the focus of material, electrical, and chemical engineers as of today. To achieve this, many fillers have been utilized to enhance the conductive ability of polymers. Polymers are simply plastics or insulators, which are macromolecules, made up of monomers in long chain and obtained through chemical reaction called polymerization [4]. The uniqueness of amalgamation of fillers with polymers is that, it enhances the behaviors of polymers (electrical, mechanical, and thermal). When nanocarbons interact with polymers, their nature is determined by the nanofiller concentrations. Some of the fillers used to improve the electrical conductivity of polymers include: carbon black, graphite, graphite oxide, graphene, carbon fiber, nanoplatelet, graphite intercalation compound. Lee et al. [5] investigated the thermal conductivity of PCs by using aluminum nitride (AlN), wollastonite, silicon carbide whisker, boron nitride. In all, these fillers, when mixed with polymers, were found to increase the electrical conductivity of polymers.

Quartz crystal microbalance method was used by [6] to fabricate electrochemical electrode for supercapacitor. The electrode is a composite of multiwalled-CNT loaded polypyrrole, doped with nitrogen. Cycle stability, high capacitance, high energy, and high power densities are some of the advantages. It is important to note that the improvement in the electrical conductivity of this conjugated conductive polymer (polypyrrole), is as a result of the introduction of MWCNT nanofiller into it. Also, in the process of fabricating electrode for electrochemical supercapacitor by [7], a cationic dye (malachite-green) was accumulated on the surface of polypyrrole-MWCNT composites. According to the author [7], the polypyrrole was synthesized by: (i) dissolving cetrimonium bromide in hydrochloric solution, (ii) adding ammonium persulfate to the solution in (i), (iii) stirring the solution in (ii) for some minutes, and (iv) adding pyrrole monomers to the solution in (iii). The step-by-step fabricating method of this electrode can be found in [7]. Spinning, sol-gel, solution casting, in-situ, electrochemical, and template synthesis are composites preparation methods [8]. Shi and Zhitomirsky [9] reported electrophoretic method for the fabrication of graphene loaded MWCNT, and graphene loaded polypyrrole. Although the investigations of [6,7,9] were experimental, the classic advantage of polymer composites in storing energy were elucidated. The tunable properties of polymers and its composites, also find advantages in water purification [6,8,10].

The combination of filler(s) and polymer matrix usually forms a network (N). The electrical conductivity of N-forms can be detected by a set of equations, known as percolation equation. The point at which the electrical conductivity of the N-forms becomes a complete short-circuiting of charges from end-to-end of the material, is called the percolation threshold [2,11,12,13]. Percolation theory have been investigated to be a viable theory, which can model electrical conductivity of composites accurately. The reason behind the electrical conductivity of polymer-composites is the percolation theory facts. By understanding percolation theory enables a thorough knowledge of electrical conductivity of polymer-composites. The point at which filler begins continuous conduction in polymer-composites, is called percolation threshold. This is very useful to sustain energy in solar devices and batteries. At percolation threshold, the conductivity of polymer-composite is of several order of magnitude. This shows that percolation threshold is the minimum concentration of filler, which results in continuous conductive pathways throughout the polymer matrix. Beyond this point, the conductivity of a polymer-composite increases at high magnitude as the filler loading increases. Researchers have identified various factors responsible for the variability of fillers in polymer matrix to be: filler purity, length, aspect ratio, size, orientation, surface energy, and matrix energy, etc.

An argument postulated by [14] is that the conductivity of polymer-composites is a function of its packing factor. The packing factor $P_{f}$ is a dependent parameter on the shape and structure formation of the particle. The relation between the packing factor and the electrical conductivity of polymer-composites, for a randomly distributed particle, is presented by Equations (1) and (2)

(1) $$\sigma_{c}=\left(\frac{\phi_{f}}{\phi_{f}+\phi_{p}}\right)X_{c}$$

(2) $$P_{f}=\left(\frac{\phi_{f}}{\phi_{f}+\phi_{p}}\right)$$

where $\sigma_{c}$ is the electrical conductivity of the polymer-composite, $\phi_{f}$ is the volume occupied by the filler, $\phi_{p}$ is the volume occupied by the polymer, $X_{c}$ is a critical parameter for models, having just a single $\sigma_{c}$ for lattice problems. Equation (1) is only suitable for randomly distributed conductive composites [15].

**Figure 1:**
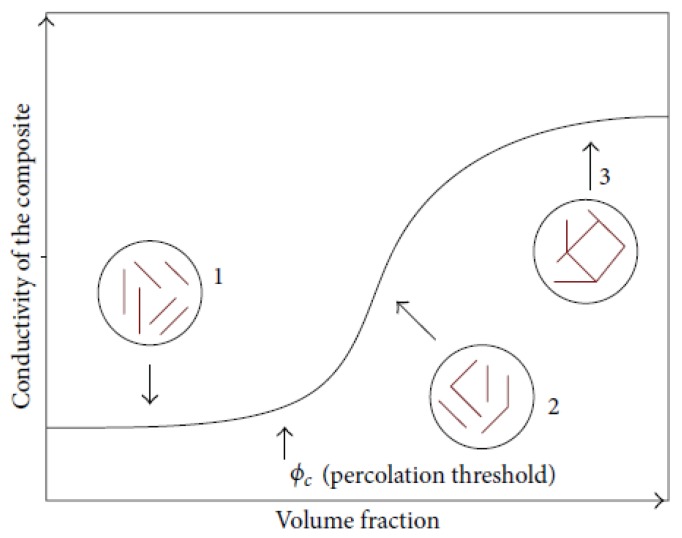
The percolation regions of conducting polymer composites [16].

Figure 1 is a graphical picture of percolation *s* like-shape. It has three regions. In region 1, the percolation threshold has not been reached, and the conductivity of the composite is approximately equal to the conductivity of the polymer matrix. In region 2, the percolation is reached and the conductivity of the composite increases at high magnitude. In region 3, the optimum electrical conductivity of the polymer-composite is reached.

The evidence of transition of insulator to conductor is proven by the percolation theory [17]. A recent model proposed by [18] was tested on carbon nanotube mixed with polydimethylsiloxane polymer with results reported to be in agreement with experimental data. The results obtained from the model and the experiment were used to manufacture sensor, which has the ability to respond to mechanical perturbation. In addition, Clingerman et al. [19] proposed a model to determine the electrical conductivity of thermocarb specialty graphite, Ni-coated polyacrylonitrile (PAN) carbon fiber, and chopped/milled forms of PAN loaded on nylon 6,6 and polycarbonate. The model is referred to by many authors as the Mamunya model. The result of the model closely agreed with the experimental data obtained. The aspect ratio and the surface energy of the filler and polymers were the parameters the model took into consideration. All the existing models rely on percolation theory. Statistic, thermodynamic, structured-orientation, and geometric, etc., are all examples of percolation theory models. There are mathematical and empirical rule models which are used to predict the electrical conductivity of polymer-composites [20]; the models must include fillers and matrix parameters, on which the final properties of the materials depend [21].

The ability to control the electrical resistance of polymers by adding additives to them, is a promising way of having substitute for silicon and germanium electronic devices [22,23]. An investigation on poly(vinyl alcohol) loaded lithium hydroxide and magnetite FeO${}_{4}$ electrolyte, was carried out by [24]. This polymer-composite electrolyte is suitable for electrochemical, and magnetic devices. Furthermore, nanocomposites of inorganic materials for health monitoring sensor was investigated by [25]. The fast electrical response of the composite when subjected to mechanical stress, revealed that the material is suitable for the manufacturing of health monitoring sensors. The benefits associated with polymer composites is enormous, therefore, pungent effort is required to research on it.

This paper studied the mathematical formulation of the recent and frequently used models, and identified the various parameters considered. Moreover, the suitability of each model is analyzed. Furthermore, a proposition is made for the modification and inclusion of more parameters on each models for various device applications.

Table 1 is the summary of the applicable areas of conductive polymer-composites [26].

## 2. The Existing Models and Their Formulation

### 2.1. Weber Models

Weber and Kamal [17] developed models which were tested on nickel-coated graphite and loaded on polypropylene. These models were named End-to-End (EEM) and Fiber Contact Model (FCM). These models belong to the group of classical percolation theory models. The models are presented as follow:

The fiber notation: length *l*, and diameter *d*

The composite sample notation: length *L*, width *W*, and thickness *T*

Two cases were assumed:

Case 1: The fiber is aligned in the test direction, and the composite electrical conductivity is proportional to the fiber electrical conductivity, i.e.,

(3) $$\sigma_{c}\propto \sigma_{f}$$

which is equivalent to:

(4) $$\sigma_{c}=\sigma_{f}n\kappa_{f}\kappa^{-1}$$

where $\sigma_{f}$ is the conductivity of fiber, $\sigma_{c}$ is the conductivity of composites, n is the number of strings of conductive fibers, $\kappa_{f}$ is the cross-sectional area of fiber string, and κ is the cross-sectional area of composite sample. Equation (4) can be written in terms of volume, i.e.,

(5) $$\sigma_{c}=\sigma_{f}n\left(\frac{\kappa_{f}L}{\kappa .L}\right)$$

The numerator $\kappa_{f}L$ is the fiber volume, while the denominator κ.L is the composite sample volume. The volume fraction, ϕ is:

(6) $$\phi =\frac{\kappa L}{n\kappa_{f}L}$$

The electrical conductivity of the polymer-composite, when aligned in test direction, is now given as $\sigma_{c}$,

(7) $$\sigma_{c}=\frac{\sigma_{f}}{\phi }$$

Case 2: When the fibers are aligned at an angle θ, the filler size then becomes a very important parameter for the prediction of the electrical conductivity of the polymer-composite. The angle changes the fiber orientation and the electrical conductivity, $\sigma_{c}$ becomes:

(8) $$\sigma_{c}=\sigma_{f}\cos^{2}\theta \phi_{p}$$

The ratio of the volume fraction is:

(9) $$\frac{\phi_{p}}{\phi }=\left(1-\frac{L}{W}\cos\theta \right)$$

The electrical conductivity along the longitudinal plane is:

(10) $$\sigma_{c_{l}ong}=\sigma_{f}\phi \cos^{2}\theta \left(1-\frac{L}{W}\tan\theta \right)$$

The electrical conductivity along the transverse direction is:

(11) $$\sigma_{c_{t}rv}=\sigma_{f}\phi \sin^{2}\theta \left(1-\frac{L}{W}cot\theta \right)$$

Equations (10) and (11) is called EEM as proposed by [17].

The Fiber Contact model considers: length, orientation, and volume fraction of polymer-composites. The model is presented as follows: for the longitudinal direction

(12) $$\rho_{c}=\frac{\pi d^{2}\rho_{f}X}{4\phi_{p}d_{c}l\cos^{2}\theta }$$

for the transverse direction

(13) $$\rho_{c}=\frac{\pi d^{2}\rho_{f}X}{4\phi_{p}d_{c}l\sin^{2}\theta }$$

where $\phi_{p}=\beta \phi$, and β is a function of the percolation threshold, $d_{c}$ is the diameter of a circle of contact, *d* is the fiber diameter, and *l* is the fiber length, *X* is a function of contact point, $\rho_{c}$ is the composite resistivity, and $\rho_{f}$ is the filler resistivity. The application of these two models to predict the electrical conductivity of nickel-coated graphite mixed with polypropylene, showed reasonable agreement with the experimental data.

### 2.2. Power Law Equation

Kirkpatrick [27] proposed the power law equation, which is a statistical percolation model and was tested to predict the electrical conductivity of materials. The equation, though effective, did not prove to be accurate when compared with experiment data [14,19]. However, when [14] conducted an experiment on the mixture of epoxy resin/(copper, nickel), and poly(vinyl chloride) with metal, the model was very efficient; this suggests that the power law equation can model randomly distributed mixture of materials with metal-like structure. The equation is presented as follows:

(14) $$\sigma_{c}\propto {\left(P-P_{c}\right)}^{t}$$

where the $\sigma_{c}$ is the conductivity of the mixture, *P* is the filler concentration, $P_{c}$ is the threshold concentration and *t* is the critical exponent, which has a value of 1.6±0.1 for bond percolation and 1.5±0.2 for site and correlated bond percolation [27]. The normalized form of Equation (14) is given by [14] as:

(15) $$\frac{\sigma -\sigma_{c}}{\sigma_{m}-\sigma_{c}}={\left(\frac{P-P_{c}}{F-P_{c}}\right)}^{t}$$

The gained conductivity, σ, of the composite is therefore, given as:

(16) $$\sigma =\sigma_{c}+\left(\sigma_{m}-\sigma_{c}\right){\left(\frac{P-P_{c}}{F-P_{c}}\right)}^{t}$$

Table 2 is the summary of the work conducted by [14], which was tested with the normalized power law equation.

From Table 2, the values of log σ can be calculated and by curve-fitting, while *t* and $\sigma_{m}$ can also be calculated. Testing the normalized Power Law Equation on the experimental values (ER-Cu, PVC-Cu, ER-Ni) of Table 2, the values were perfectly matched. The deduction from this observation is that the percolation theory can be further worked upon and a promising output is evidently assured for some composites.

### 2.3. Eight-Chain Model

Jang and Yin [18] developed a model to predict the electrical conductivity of multi-walled carbon nanotube (MWCNT) loaded on polydimethylsiloxane. The model was said to be useful for the design and analysis of some composites which has ability to respond to impulse. The final output is to manufacture displacement sensors. The idea of [18] was based on the elementary understanding of resistivity as given in Equation (17).

(17) $$\sigma_{eff}=\frac{1}{l_{u}R_{f}}$$

where $l_{u}$ is the composite length, $R_{f}$ is the resistance from point to point. Due to the waviness of MWCNT, the effective length was modeled as $l_{f}$:

(18) $$l_{f}=\sqrt{3}\Lambda \Gamma^{\frac{1}{2}}\sqrt{\left(\frac{1+\cos\vartheta }{1-\cos\vartheta }\right)}$$

Λ is the bond distance, Γ is the layer of filler-to-filler, and ϑ is the angle between the composite layers. In addition, the intrinsic resistance of the filler is $R_{int}$:

(19) $$R_{int}=\frac{4\lambda }{\pi Y^{2}\sigma_{int}}$$

where $\sigma_{int}$ is the intrinsic resistance of the conductive filler, Y is the filler diameter, and λ is the filler point-to-point length. The tunneling effect gives a point-to-point resistance $\Omega_{c}$, which was modeled as:

(20) $$\Omega_{c}=\frac{h^{2}\tau }{jq^{2}{\left(2mh_{v}\right)}^{\frac{1}{2}}}exp\left(\frac{4\pi \tau }{h}{\left(2mh_{v}\right)}^{\frac{1}{2}}\right)$$

where j is the point-to-point area which is approximately equal to $Y^{2}$, *q* is the electronic charge, *h* is Planck’s constant, τ is the carbon-to-carbon distance, which is a function of the volume fraction, *m* is the mass of electron and $h_{v}$ is the potential barrier height. Jang and Yin [18] concluded that these equations were used to predict the electrical conductivity of MWCNT/PDMS with high accuracy and in agreement to experimental data. The validity of the conclusions made by [18], have not been verified by any researcher as of present.

### 2.4. McLachlan (GEM) Model

McLachlan et al. [28] combined Bruggeman and Percolation theories together to produce the General Effective Model (GEM). This model has been investigated by many researchers with comments of good commendations [11,19]. The general conductivity equation, by following the classical percolation theory, is given as:

(21) $$\sigma_{m}=\sigma_{h}\left(1-\frac{\nabla }{\nabla_{c}}\right)$$

where $\sigma_{m}$ is the conductivity, $\sigma_{h}$ is the conductivity of the highly conductive phase, *∇* is the volume fraction of the poorly conductive phase, $\nabla_{c}$ is the critical percolation volume. Avoiding complex derivation, the General Effective Model equation is given as:

(22) $$\frac{\nabla (\sigma_{l}^{1/t}-\sigma_{m}^{1/t})}{\sigma_{l}^{1/t}+X\sigma_{m}^{1/t}}+\frac{\left(1-\nabla \right)\left(\sigma_{h}^{1/t}-\sigma_{m}^{1/t}\right)}{\sigma_{h}^{1/t}+X\sigma_{m}^{1/t}}=0$$

where *t* is the percolation and the GEM exponent, *X* is a constant, which can be deduced from experimental data. For instance, when $\sigma_{l}$ is set to zero, then Equation (22) is reduced to percolation model, as shown:

(23) $$\begin{array}{c} \frac{\nabla }{X}+\frac{\left(1-\nabla \right)\left(\sigma_{h}^{1/t}-\sigma_{m}^{1/t}\right)}{\sigma_{h}^{1/t}+X\sigma_{m}^{1/t}}=0 \end{array}$$

(24) $$\begin{array}{c} \sigma_{m}^{1/t}-\sigma_{h}^{1/t}\left(1-\frac{\nabla }{\nabla }\right)=0 \end{array}$$

By comparing Equations (21) and (23), it is immediately seen that they are the same. *t* is defined thus:

(25) $$\frac{1}{1-(\gamma_{f}-\gamma_{\phi })}$$

where $\gamma_{f}$ is the coefficient characterizing the low-conductive phase, and $\gamma_{\phi }$ is the coefficient characterizing the high-conductive phase. The GEM model was not derived, therefore its efficiency can only be based on limits of the composites, and observing how well the equation models the electrical conductivity [28].

### 2.5. Modified McLachlan (GEM) Model

Kakati et al. [29] reported that the original version of GEM, models sufficiently well, the electrical conductivity of polymer-composites with regular shape but it has the following short-comings:

i The modeling of the electrical conductivity of polymer-composite becomes difficult when the conductivity of both materials are widely apart.
ii The model did not consider the in-plane and through-plane conductivity, therefore modeling of a network with composites such as carbon black and natural graphite, probably, will never be accurate.

**Figure 2:**
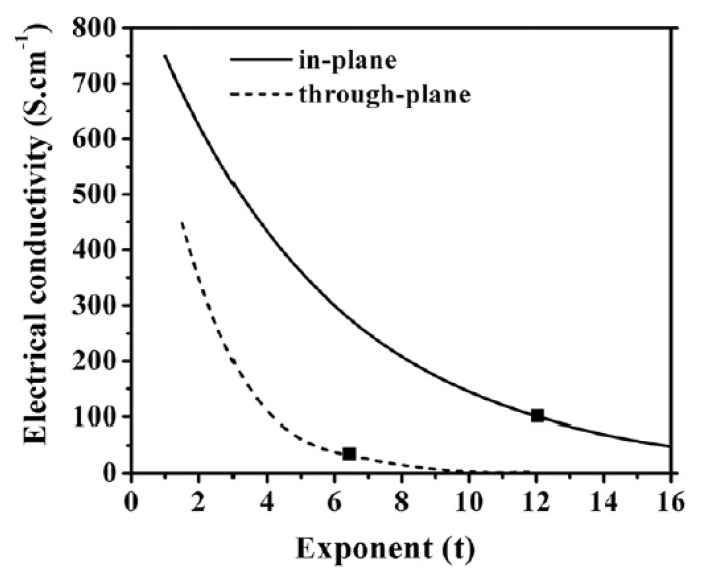
Exponential curve of the electrical conductivity of a 60:40 ratio mixture of NG/PF [29].

Figure 2 shows an important parameter in GEM and that is the critical exponent, *t*. The electrical conductivity of the mixture of natural graphite and phenol-formaldehyde decreases exponentially as the critical exponent, *t*, changes, which is defined by the equation:

(26) $$\begin{array}{c} t=xe^{y\nabla } \end{array}$$

where *x* and *y* are composite network constants. The modified general effective model (MGEM) is given by the equation:

(27) $$\sum_{j}^{N}\frac{\nabla_{j}\left(\sigma_{j}^{1/t}-\sigma_{m}^{1/t}\right)}{\sigma_{j}^{1/t}-X\sigma_{m}^{1/t}}=0$$

Equation (27) conquered the limitations experienced as a result of orientation and shape factors in determining the electrical conductivity of polymer-composites [29].

### 2.6. Mamunya Model

The PVC-Ni composite concentration, σ, of the research conducted by Mamunya et al. [14] did not obey the normalized power law equation of Equation (16). The reason for this is that the Power Law (Percolation equation) cannot solve non-statistical problems. Therefore, Mamunya et al. [14] took the logarithm of Equation (16), which yielded what is referred to as the Mamunya Model. The model was used to verify the conductivity of PVC-Ni and the result showed consistency with the experimental data. The model is presented as follows

(28) $$log\sigma =log\sigma_{c}+\left(log\sigma_{m}-log\sigma_{c}\right){\left(\frac{P-P_{c}}{F-P_{c}}\right)}^{k}$$

where *k* is a replacement of *t* in Equation (16) which can be calculated from:

(29) $$k=\frac{KP_{c}}{{\left(P-P_{c}\right)}^{0.75}}$$

where *K* can be calculated from the following equation

(30) $$K=0.28-0.036\delta_{pf}$$

$\delta_{pf}$ is the interfacial surface tension. The interfacial surface tension is the value which can be determined from Equation (31).

(31) $$\delta_{pf}=\delta_{p}+\delta_{f}-2{\left(\delta_{p}\delta_{f}\right)}^{0.5}$$

where $\delta_{p}$ is the polymer surface tension, $\delta_{f}$ is the filler surface tension [30].

### 2.7. Clingerman Additive Model

The essence of the Clingerman Additive Model was to create a set of mathematical equations that will model the electrical conductivity of polymer-composites with different properties [31,32]. While formulating the equation, the standard mixing rule is shown as follows:

(32) $$\sigma =\sum \phi_{i}\sigma_{i}$$

$\phi_{i}$ is the volume of component *i*, and $\sigma_{i}$ is the conductivity of the component *i*. The model cannot predict accurately the electrical conductivity of polymer-composites [31]. The reason for this is because the equation did not include percolation threshold. The form of equation that will predict electrical conductivity of PCs is given as follows:

(33) $$log\sigma =\left\{\begin{array}{cc} log\sigma_{p} & \mathrm{for}\;\phi \leq \phi_{c}\\ log\sigma_{p}+log\sigma_{f}{\left(\phi -\phi_{c}\right)}^{t}\pm & \mathrm{for}\;\phi >\phi_{c}\\ f\left(struc\right)\pm f\left(\delta_{pf}\right) \end{array}\right.$$

Equation (33) accounts for different factors, which include: interfacial tension $\delta_{pf}$, filler conductivity $\sigma_{f}$, filler volume fraction σ, percolation threshold $\sigma_{c}$, and critical exponent *t*. The term $log\sigma_{f}{\left(\phi -\phi_{c}\right)}^{t}$ accounts for the effect of filler conductivity for a volume fraction above the percolation threshold, the term f(struc) accounts for the structure of the composite materials, and $f(\delta_{pf})$ accounts for polymer–filler interaction. Based on the aspect ratio, [33] postulated an equation that describes the shape of the particle. The shape factor, h(a) is given as:

(34) $$h\left(a\right)=\left\{\begin{array}{cc} \left(A^{2}(1-0.5\left(A-\frac{1}{A}\right)In\left(\frac{A+1}{A-1}\right)\right) & \mathrm{for}\;1<a<\infty \\ 1 & \mathrm{for}\;a\to \infty \end{array}\right.$$

where *A* is a variable, which depends on the aspect ratio. The value of $A^{2}$ is:

(35) $$A^{2}=\frac{a^{2}}{a^{2}-1}$$

where *a* is the aspect ratio. This shape factor forms the basis for the structure term. The f(struc) is given by the equation:

(36) f(struc)=h(a)∗cos(ω)

where ω is the orientation angle, which its cosine increases as the conductivity increases. The surface tension is expected to reduce for the conductivity to increase, therefore, the surface energy term is given as:

(37) $$f\left(\delta_{pf}\right)=-Y\delta_{pf}$$

*Y* is a constant that can be obtained by curve-fitting. The critical exponent *t* is also given as:

(38) $$t=\frac{U\phi_{c}}{{\left(\phi -\phi_{c}\right)}^{n}}$$

where *n* and *U* are constants that can be obtained by regression analysis. By introducing a scaling factor filler terms in the Equation (33) and combing all the terms, the following equation is obtained for the Additive Electrical Model of Polymer-Composites (AEMPCs).

(39) $$log\sigma =\left\{\begin{array}{cc} log\sigma_{f} & \mathrm{for}\;\phi \leq \phi_{c}\\ log\sigma_{p}+Dlog\sigma_{f}{\left(\phi -\phi_{c}\right)}^{\frac{U\phi_{c}}{\phi -\phi_{c}}}... & \mathrm{for}\;\phi >\phi_{c}\\ \;+h\left(a\right)\cos\left(\omega_{pf}\right) \end{array}\right.$$

where *D* is the scaling factor.

### 2.8. Monte Carlo Method

**Figure 3:**
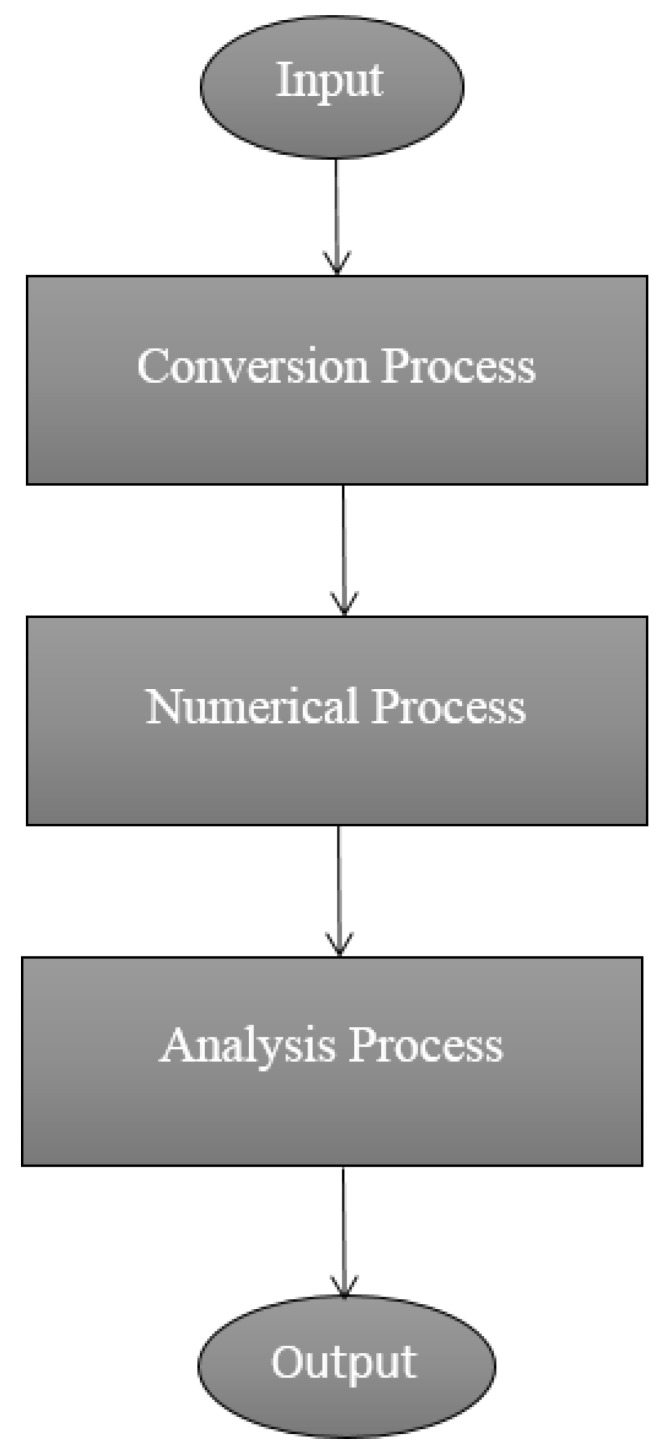
Monte Carlo method flow chart process.

The computational algorithm is the algorithm that calculates the sharing of unknown probabilistic entity by continuous random sampling [34]. The Monte Carlo method is a statistical model that generates random position parameters and shape parameters of polymer-composites [20,35]. This method usually involves three processes; the processes are presented in Figure 3. The conversion process takes the physical problem and translates it to a statistical model, the numerical process solves the statistical problem by using suitable statistical solver and the analysing process obtains the data from stage two and deduces the system properties.

**Figure 4:**
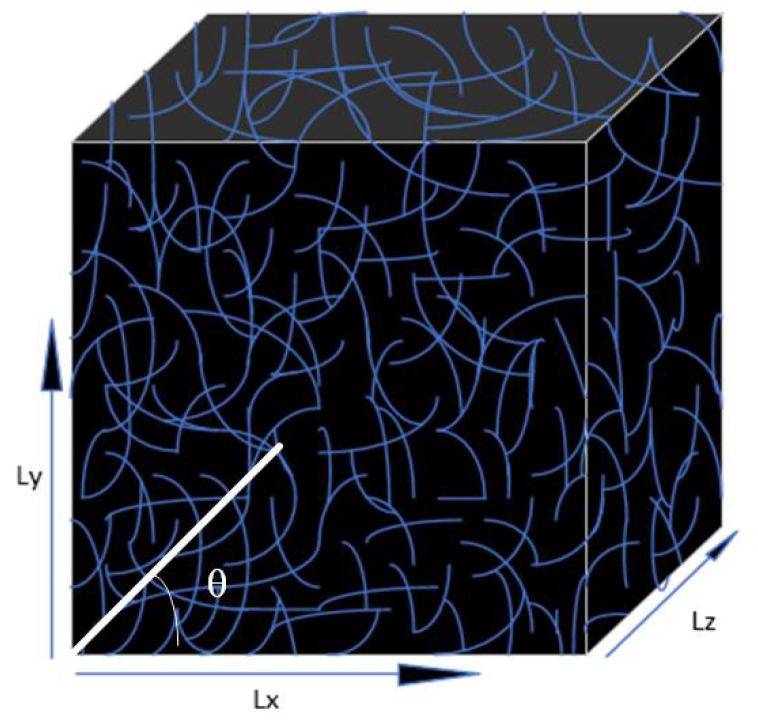
Typical composite: randomly moving in a Representative Volume Element (RVE) with orientation angle θ.

According to [36], Monte Carlo simulations are performed to show how the following parameters relate to conductivity: (1) volume fraction, (2) fiber solidity, (3) fiber aspect ratio, (4) coating layer thickness, and (5) fiber orientation angle. To illustrate Monte Carlo method for electrical conductivity determination of PCs, it is important to demonstrate the soft-core model of Figure 4. Figure 4 is a 3D soft core model (3D mean 3-plane, while the soft core mean fibers are bonded when overlapped and the model is fully interpenetrating [37]).

If the starting point and the end point of particle-to-particle is $x_{j1},y_{j1},z_{j1}$, $x_{j2},y_{j2},z_{j2}$, then the equations representing the starting point coordinate is given in Equations (40)–(42).

(40) $$\begin{array}{c} x_{j1}=L_{x}.rnd \end{array}$$

(41) $$\begin{array}{c} y_{j1}=L_{y}.rnd \end{array}$$

(42) $$\begin{array}{c} z_{j1}=L_{z}.rnd \end{array}$$

The end point coordinates are:

(43) $$\begin{array}{c} \left(\begin{array}{c} x_{j2}\\ y_{j2}\\ z_{j2} \end{array}\right)=\left(\begin{array}{c} x_{j1}\\ y_{j1}\\ z_{j1} \end{array}\right)+L_{f}\left(\begin{array}{c} \cos(2\pi .rnd)\\ \sin(2\pi .rnd)\\ 1 \end{array}\right)\left(\begin{array}{c} \sin\theta_{j}\\ \sin\theta_{j}\\ \cos\theta_{j} \end{array}\right) \end{array}$$

(44) $$\cos\theta_{j}=\left(1-\cos\theta_{h}\right).rnd+\cos\theta_{h}$$

where $L_{f}$ is the length of fiber, $\cos\theta_{h}=\frac{\pi }{2}$ for non-preferential orientation and $\cos\theta_{h}=0$ for composites horizontal alignment in the direction of current flow [38]. The total resistance in the composites network is:

(45) $$\begin{array}{c} R=R_{pure}+R_{c} \end{array}$$

$R_{pure}$ is the intrinsic resistance of the composites, $R_{c}$ is the contact resistance between sheets of composites. The contact resistance is defined as:

(46) $$R_{c}=\frac{h}{2q^{2}MT}$$

and

(47) $$R_{pure}=\frac{4l_{jk}}{\sigma_{pure}\pi D^{2}}$$

where $l_{jk}$ is the contact point length, $\sigma_{pure}$ is the intrinsic conductivity if the composites, *D* is the composite thickness (if it is not circular), *M* is the number of conduction channels, *q* is the charge on electron, *T* is charge transmission probability, and *h* is Planck’s constant. The conductivity of the polymer-composite is:

(48) $$\sigma_{t}=\frac{L_{x}}{R_{eqv}L_{y}L_{z}}$$

where $\sigma_{t}$ is the conductivity derived with respect to Monte Carlo method [39,40,41].

### 2.9. Maxwell Equation

In 1873, Maxwell postulated an equation for an infinitely diluted composite of spherical filler particles [42,43]. The expression is presented as follows:

(49) $$\begin{array}{c} \frac{\sigma }{\sigma_{m}}=1+3\phi \left(\frac{\sigma_{d}-\sigma_{m}}{\sigma_{d}+2\sigma_{m}}\right) \end{array}$$

where σ is the electrical conductivity of composite, $\sigma_{m}$ is the polymer conductivity, $\sigma_{d}$ is the conductivity of filler dispersed-phase, and ϕ is the volume fraction of filler. Reducing Equation (49) to a simpler form, Equation (50) is derived.

(50) $$\sigma_{1/m}=1+3\phi \left(\frac{\sigma_{d/m}-1}{\sigma_{d/m}+2}\right)$$

where $\sigma_{1/m}$ is relative electrical conductivity, which is equivalent to $\frac{\sigma }{\sigma_{m}}$ and $\sigma_{d/m}$ is the electrical conductivity ratio, which is equivalent to $\frac{\sigma_{d}}{\sigma_{m}}$. In order to test the functionality of the equations, the following expression is made:

As $\sigma_{d/m}\to 0$, then

(51) $$\sigma_{1/m}=\left(1-\frac{3}{2}\phi \right)$$

and as $\sigma_{d/m}\to \infty$

(52) $$\sigma_{1/m}=1+3\phi$$

### 2.10. Maxwell–Wagnar Equation

The Maxwell–Wagner model caters for high concentration of fillers [20,42]. It is an extension of the Maxwell equation by Wagner. The equation shows that conductivity is directly proportional to volume fraction of fillers. The following expressions provide Maxwell–Wagner equations.

(53) $$\left(\frac{\sigma -\sigma_{m}}{\sigma +2\sigma_{m}}\right)=\left(\frac{\sigma_{d}-\sigma_{m}}{\sigma_{d}+2\sigma_{m}}\right)\phi$$

which can be expressed as:

(54) $$\left(\frac{\sigma_{1/m}-1}{\sigma_{1/m}+2}\right)=\left(\frac{\sigma_{d/m}-1}{\sigma_{d/m}+2}\right)\phi$$

Further simplifying Equation (54), the following expression is derived:

(55) $$\sigma_{1/m}=\frac{1+\frac{2\phi (\sigma_{d/m}-1)}{\sigma_{d/m}+2}}{1+\frac{\phi (1-\sigma_{d/m})}{\sigma_{d/m}+2}}$$

As $\sigma_{d/m}\to 0$, then

(56) $$\sigma_{1/m}=1$$

and as $\sigma_{d/m}\to \infty$, then

(57) $$\begin{array}{c} \sigma_{d/m}=\frac{1+2\phi }{1+\phi } \end{array}$$

The deduction from both the Maxwell and Maxwell–Wagner equations is that the electrical conductivity of a polymer-composite is directly proportional to the volume fraction of the fillers.

### 2.11. Pal Model

Maxwell model is constrained to solve spherical filler shape composites [42]. However, to the generalized Maxwell equation, Pal applied a correcting factor to the Equation (49), so that the electrical conductivity of particulate composites with non-spherical shape fillers can also be predicted. The equation is as expressed:

(58) $$\frac{\sigma }{\sigma_{m}}=1+3\epsilon \left(\frac{\sigma_{d}-\sigma_{m}}{\sigma_{d}+2\sigma_{m}}\right)\phi$$

Equation (58) is known as the Generalized Maxwell equation, where ϵ is the correcting factor. Pal performed a long mathematical derivations in order to arrive at the final model. The summary of the expression is as follows:

(59) $${\left(\sigma_{1/m}\right)}^{1/3}\left(\frac{\sigma_{d/m}-1}{\sigma_{d/m}-\sigma_{1/m}}\right)=\left(1-\frac{\phi }{\phi_{m}}\right)$$

As $\sigma_{d/m}$→*∞*, then

(60) $$\begin{array}{c} \sigma_{1/m}={\left(1-\frac{\phi }{\phi_{m}}\right)}^{-3\epsilon \phi_{m}} \end{array}$$

Also, as $\sigma_{d/m}\to =0$, then

(61) $$\sigma_{1/m}={\left(1-\frac{\phi }{\phi_{m}}\right)}^{\frac{3\epsilon \phi_{m}}{2}}$$

where $\phi_{m}$ is the matrix volume fraction. Equation (60) can be reduced to the Bruggeman model, if ϵ=1 and $\phi_{m}=1$, i.e.,

(62) $$\begin{array}{c} \sigma_{1/m}={\left(1-\phi \right)}^{-3} \end{array}$$

### 2.12. Sigmoidal Function

**Figure 5:**
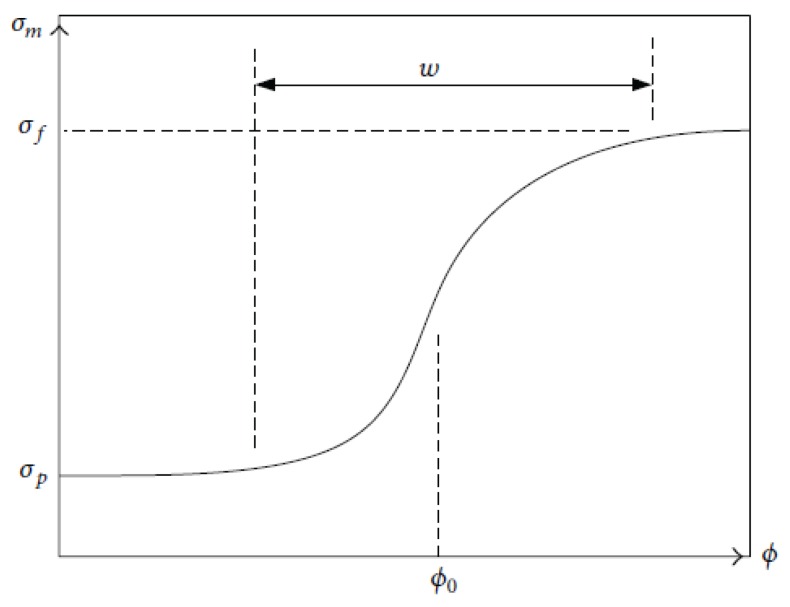
Simoidal function for predicting electrical conductivity of polymer-composites [16].

There are several sigmoidal functions as presented by [44,45], which include logistic function, Gompertz model, extreme value function, Chapman-Richards, cumulative Weibull distribution, Hill function, Lomolino function, cumulative beta-P distribution. The logistic function given in Equation (63) has been tailored to predict the electrical conductivity of nanocomposites [16].

(63) $$f\left(x\right)=\frac{a}{1+\exp(-bx+c)}$$

where a,b,c are constants, and f(x) is the dependent value. The sigmoidal predictor model is given in Equation (64).

(64) $$\sigma_{m}=\sigma_{p}+\frac{\sigma_{f}-\sigma_{f}}{1+\exp\left(\frac{-1+\phi_{mp}}{w}\right)}$$

where *w* is the width of the region of percolation, as shown in Figure 5. Comparing Equations (63) and (64), the constants of the Logistic function as related to nanocomposite conductivity predictor, are: $a=\sigma_{f}-\sigma_{f}$, b=1/w and $c=\frac{\phi_{mp}}{w}$

## 3. Factors Affecting the Electrical Conductivity of Polymer Composites

**Figure 6:**
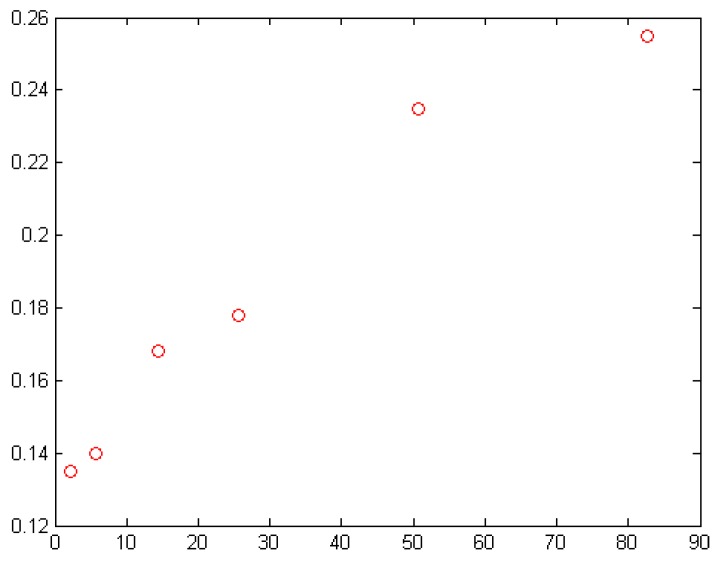
Particle size versus volume fraction [47].

A remarkable conclusion was given by [46] on the basis of concentration, filler particles types, and average size as factors which affect the electrical conductivity of polymer composites. For nanocomposite models to be fully explored, many parameters need to be involved in the selected models. Conductivity (filler, matrix, and at maximum packing factor, at percolation), and critical exponent and width of the percolation, have to be considered [16]. Table 3 is a summary of the parameters of the models discussed in this work. The target is the percolation threshold. How does this individual parameter affect the percolation threshold in terms of volume fraction? How can percolation threshold be achieved at minimum volume fraction of fillers? Answers to these questions will pave the way for a better model and design of electrical conductivity of polymer composite devices for many applications. The effect of size and shape of the filler on the electrical conductivity of polymer composites is also very consequential. For plate-like graphite loaded on low density polyethylene [47], it was observed that the volume fraction of the filler increased linearly as the mean particle size increased. This relationship is presented in Figure 6. The particle shape depicted in Figure 6 is spherical; carbon black, as an example of spherical particle, finds it very difficult to create conducting channels as the diameter increases [1].

Chen et al. [54] studied the possible connections of hybrid carbon-black and carbon nanotube using Monte Carlo simulation: volume fraction and dimensions of the fillers were the concerned parameters. In the study, it was discovered that increase in filler diameter, and aspect ration, reduces electrical conductivity of polymer composites. The focus of many of the models was connected to additives volume fraction effect on the electrical conductivity of composite materials: percolation depends upon so many more factors than just volume fraction. The omitted parameters include the nature of polymer matrices and filler, which could alter the percolation of the composites [55]. Pisitsak et al. [56] explained how viscosity and shear rate affects the electrical conductivity of polymer composites of carbon nanotube loaded liquid crystalline polymer: the same volume ratio of additives mixed with different polymers yielded different electrical conductivity values. Singh et al. [57] determined the electrical conductivity of ABS-Graphene experimentally, using Ohms law; volume fraction and manufacturing method were reported to have been the factors responsible for the variation in the sample conductivity. Also, through an experiment conducted by [58], filler orientation of carbon fiber-loaded polypropylene was said to be the predominant factor responsible for the sample electrical conductivity. When [58] applied the modified fiber contact model to predict CF/polypropylene, the results showed wide deviation from the experimental values: the reason for this is that insufficient parameters were used to characterize the model. The Mamunya model was compared with the sigmoidal-Boltzmann model of Equation (64), by [55], and the conclusion was that the sigmoidal-Boltzmann model is more accurate than the Mamunya model.

Another parameter to discuss is the aspect ratio. Aspect ratio is the ratio of the length of the particle to its width. Aspect ratio, predominantly, determines the electrical conductivity of polymer-composites. As the filler aspect ratio increases, the particles produce enough flow of current density per field developed when they disperse in the polymer matrix. As the aspect ratio increases, the volume fraction decreases, thereby increasing the electrical conductivity. Simply put, high aspect ratio lowers the percolation threshold. Table 4 shows how the aspect ratios of some materials determine their conductivities. Shape and surface layer, can be adjusted to improve thermal conductivity of the composites [59].

## 4. Conclusions

The criteria for selecting models for the prediction of the electrical conductivity of polymer-composites have been discussed. All the models are unique, but none of them can be applied as a general purpose model for the electrical conductivity of nanofiller/polymer composites. To the best of our knowledge, this review has been critically carried out. The various existing models need modifications and more experiments to be conducted. Orientation factor may not predominantly affect the conductivity of graphene/polydimethylsiloxane, while it may be the major factor for carbon-black/polydimethylsiloxane. Variation of these parameters determines the application of the materials. The various underlining parameters which affect electrical conductivity of polymer-composites should first be understood before embarking on the application of any of the models. The criterium for selecting a model that best predicts the electrical conductivity of polymer-composites is one that has all the defined parameters that determine the electrical conductivity of the filler–polymer network. Of course, there is no such model in the literature as of yet. The closest models suitable for the prediction of the electrical conductivity of polymer-composites are the General Effective Media and Monte Carlo simulations. A multi-parametric model is therefore suggested for the prediction of the electrical conductivity of polymer-composites. A high precision electrical conductivity model can only be achieved with the inclusion of the chemical and internal behaviors of the composites.

## Figures and Tables

**Table 1:** Energy level applications in conductive polymer-composites.

Application	Energy State	Resistivity Value (Ω-cm)
As conductors:	Highly Conductive	$10^{-6}$–10
i. Transistors		
ii. Bipolar plates		
iii. Thermoelectric plates		
iv. Busbars etc.		
As Sensors and EMI	Conductive	10–$10^{6}$
i. Displacement sensors		
ii. Current sensor		
iii. Voltage sensors		
iv. Temperature sensors		
v. Organic liquid sensor		
etc		
Electroplating	Insulator/Conductive	$10^{6}$–$10^{11}$
i.Fuel tank		
ii. Anti-static storage tank		
iii. Mining pipes		
iv. Storage containers		
etc.		
Perfect insulator	Insulator	$10^{11}$–$10^{16}$
i. Electric cable insulator		
etc.		

**Table 2:** Experimental composite characteristics values [14].

Comp-Site	log $\sigma_{p}$ (S/m)	log $\sigma_{c}$ (S/m)	log $\sigma_{m}$ (S/m)	$\phi_{c}$	F	t
ER-Cu	−12.8	−12.5	5.2	0.05	0.30	2.9
PVC-Cu	−13.5	−13.2	5.8	0.05	0.30	3.2
ER-Ni	−12.8	−12.0	4.8	0.09	0.51	2.4
PVC-Ni	−13.5	−13.3	4.5	0.04	0.25	

**Table 3:** Parameters concerned for the models discussed.

S/N	Model	Parameters	Short-Coming	Filler/Matrix	Reference
		Shapes, orientation,			[17]
	Weber	fiber concentration,	Degree of orientation	MCF/PP, Graphite/epoxy,	[13]
1	(FCM, and MFCM)	average length	difficult to measure	CF/PP-P	[11]
			It does not account for		
			for i. particle shape,		[30]
		Carrier tunneling	ii. Interaction between	Silver-filled polymer,	[48]
2	Power Law	and critical exponent	polymer and filler	Nb-alumina	[49]
			It has not being fully		
3	Eight Chain	Volume fraction	investigated by researchers.	MWCNT/PDMS	[18]
					[2]
		Volume fraction, shape,	Unable to predict EC of CF/PP		[48]
		size, aspect critical	due to orientation at the	CF/PP, CB/PVC,	[11]
4	McLachlan (GEM)	value, orientation angle	transverse direction.	PPy/PMMA	[28]
		Critical exponential,			
		orientation and	Insufficient defined	CB,CF	[29]
5	Modified McLachlan	shape of filler	parameters	Pheno formaldehyde/NG,	[48]
		Packing factor, aspect ratio,		CF/nylon 6,6-polycarbonate,	[48]
		surface energy of Filler and	Insufficient defined	CB/polymer,	[14]
6	Mamunya	polymer, particle shape	parameters	Cu/ER-PVC, Ni-ER-PVC, PPy/PMMA	[19]
					[32]
		Surface energy	Not suitable for		[19,31]
7	Clingerman	and geometry of filler	multifiller system	CB/PMMA	[2]
		Contact resistance,			
		Alignment angle,			
		geometrical parameters,	Not suitable for	MWCNT/polymer, MWCNT/PDMS,	[50,51]
8	Monte Carlo	dispersed conductivity	multifiller system	CNT/polymer, Ag/epoxy	[51]
			Not suitable for		
9	Maxwell	Filler diameter	multifiller system	Various polymer-composites	[52]
			Not suitable for		
10	Maxwell–Wagner	Particle size	multifiller system	Various polymer-composites	[42]
		Volume fraction, conductivity	Not suitable for		
11	Pal	of filler and polymer	multifiller system	Various polymer-composites	[42]
		filller, polymer			
		conductivity, and filler	Fitting the model is	EVA/carbon fiber,	[16]
12	Sigmoidal	volume fraction	quite challenging	NBR/carbon black	[53]

**Table 4:** Relationship between aspect ratio and conductivity [60].

Material	Aspect Ration	Resistivity Ω cm
VGCF	350–650	55
xGnP-1	100	100
PAN	24	1400

## Abbreviations

The following abbreviations are used in this manuscript:

PC	Polymer composite
CPC	Conductive polymer composite
AIN	Aluminum nitride
N	Network
PAN	Polyacrylonitride
FeO${}_{4}$	Ferrate
EMI	Electromagnetic inference
EEM	End-to-end model
FCM	Fiber contact model
MWCNT	Multiwalled carbon nanotube
PDMS	Polydimethylsiloxane
GEM	General effective model
f(struc)	Structural function
AEMPC	Additive electrical model of polymer composites
PVC-Ni	Polyvinyl chloride loaded nickel
MCF/PP	Milled carbon fiber loade polypropylene
CF/PP	Carbon fiber loaded polypropylene
MWCNT/PDMS	Multiwalled carbon nanotube loaded Polydimethylsiloxane
CF/PP	Carbon fiber loaded polypropylene
CB/PVC	Carbon black loaded polyvinyl chloride
PPy/PMMA	Polypyrrole loaded Polymethymethacrylate
ABS	Acrylonitrile butadiene styrene
Cu/ER-PVC	Copper loaded epoxy resin and polyvinyl chloride
Ni/ER-PVC	Nickel loaded epoxy resin and polyvinly chloride
